# Antibacterial therapeutics for the treatment of chytrid infection in amphibians: Columbus’s egg?

**DOI:** 10.1186/1746-6148-8-175

**Published:** 2012-09-25

**Authors:** Mariska Muijsers, An Martel, Pascale Van Rooij, Kris Baert, Griet Vercauteren, Richard Ducatelle, Patrick De Backer, Francis Vercammen, Freddy Haesebrouck, Frank Pasmans

**Affiliations:** 1 Department of Pathology, Bacteriology and Avian Diseases, Faculty of Veterinary Medicine, Ghent University, Salisburylaan 133, Merelbeke B-9820, Belgium; 2 Medicem NV, Industriepark West 68, Sint Niklaas, B-9100, Belgium; 3 Department of Pharmacology, Pharmacy and Toxicology, Faculty of Veterinary Medicine, Ghent University, Salisburylaan 133, Merelbeke B-9820, Belgium; 4 Royal Zoological Society of Antwerp, Centre for Research and Conservation, Koningin Astridplein 26, Antwerpen, B-2018, Belgium

## Abstract

**Background:**

The establishment of safe and effective protocols to treat chytridiomycosis in amphibians is urgently required. In this study, the usefulness of antibacterial agents to clear chytridiomycosis from infected amphibians was evaluated.

**Results:**

Florfenicol, sulfamethoxazole, sulfadiazine and the combination of trimethoprim and sulfonamides were active *in vitro* against cultures of five *Batrachochytrium dendrobatidis* strains containing sporangia and zoospores, with minimum inhibitory concentrations (MIC) of 0.5-1.0 μg/ml for florfenicol and 8.0 μg/ml for the sulfonamides. Trimethoprim was not capable of inhibiting growth but, combined with sulfonamides, reduced the time to visible growth inhibition by the sulfonamides. Growth inhibition of *B. dendrobatidis* was not observed after exposure to clindamycin, doxycycline, enrofloxacin, paromomycin, polymyxin E and tylosin. Cultures of sporangia and zoospores of *B. dendrobatidis* strains JEL423 and IA042 were killed completely after 14 days of exposure to 100 μg/ml florfenicol or 16 μg/ml trimethoprim combined with 80 μg/ml sulfadiazine. These concentrations were, however, not capable of efficiently killing zoospores within 4 days after exposure as assessed using flow cytometry. Florfenicol concentrations remained stable in a bathing solution during a ten day period. Exposure of *Discoglossus scovazzi* tadpoles for ten days to 100 μg/ml but not to 10 μg florfenicol /ml water resulted in toxicity. In an *in vivo* trial, post metamorphic *Alytes muletensis*, experimentally inoculated with *B. dendrobatidis,* were treated topically with a solution containing 10 μg/ml of florfenicol during 14 days. Although a significant reduction of the *B. dendrobatidis* load was obtained, none of the treated animals cleared the infection.

**Conclusions:**

We thus conclude that, despite marked anti *B. dendrobatidis* activity *in vitro*, the florfenicol treatment used is not capable of eliminating *B. dendrobatidis* infections from amphibians.

## Background

Amphibian medicine is a relatively young veterinary discipline and, so far, few studies have been carried out in order to develop evidence-based treatment protocols. Current treatment protocols against amphibian pathogens are most often based on empirical evidence and lack well supported scientific evidence. This major gap in veterinary science has garnered attention with the global emergence of the deadly fungal infection chytridiomycosis, a disease linked to mass mortalities and extinctions of amphibian species during the last decades [1,2]. The causative agent, *Batrachochytrium dendrobatidis*, is an aquatic pathogen with two life stages: a uniflagellated motile zoospore and an immotile reproductive zoosporangium [3]. In infected amphibians, *B. dendrobatidis* zoosporangia are found in the upper layers of the epidermis, causing hyperkeratosis and excessive shedding of the skin [1]. In more severe cases, *B. dendrobatidis*’ capacity to disrupt normal regulatory skin functions (e.g. exchange of respiratory gases, water and electrolytes) causes electrolyte depletion and osmotic imbalance inducing clinical signs, like dehydration and anorexia, and death [4,5].

Controlling this disease and stabilizing populations of endangered amphibian species in captivity and in the wild has become a priority in amphibian conservation. The importance of clinical trials to develop an effective and safe treatment for chytridiomycosis has recently been put forward by Berger et al. [6]. At this moment minimum inhibitory concentrations *in vitro* against *B. dendrobatidis* have been determined for 10 antifungal agents: benzalkonium chloride (0.78 μg/ml), povidone iodine (312.5 μg/ml), amphotericin B (3.125 μg/ml), fluconazole (1.56 μg/ml), itraconazole (1.56 μg/ml), enilconazole (1.56 μg/ml), mercurochrome (6.25 μg/ml), sodium chloride (12.5 mg/ml), voriconazole (0.0125 μg/ml) and caspofungin (16 μg/ml) [7-9]. The use of these agents on a large scale in amphibians is hindered mainly due to toxic side effects, high prices or *in vivo* failure of activity [7-10]. Benzalkonium chloride, amphotericin B and fluconazole failed to clear *B. dendrobatidis in vivo*[7,8]. Moreover, amphotericin B was acutely toxic to *Alytes muletensis* tadpoles [8]. Formaldehyde and malachite green, even though found useful by Parker et al. [11], are extremely toxic, especially to tadpoles [12].

At present, especially itraconazole and voriconazole are antifungal agents of choice [8,10]. However, the treatment schedule of itraconazole is laborious and depigmentation of treated tadpoles has been observed [10]. Voriconazole, while apparently safe and effective, is quite expensive, the intravenous formulation hard to obtain and moreover, it is considered vital for the treatment of human patients with e.g. aspergillosis and therefore not the drug of choice for high scale use in veterinary medicine [8].

In 2009, Bishop et al. discovered the efficacy of chloramphenicol in the treatment of chytridiomycosis [13]. However, chloramphenicol is known to cause bone marrow toxicity in humans [14] and can also induce leukemia in amphibians [15]. The discovery that an antibacterial compound can be effective against *B. dendrobatidis* offers new opportunities for the development of a treatment protocol.

The aim of this study was to evaluate the usefulness of 10 antimicrobial agents for the treatment of chytridiomycosis. The minimal inhibitory concentrations were determined to make a first selection of *in vitro* efficacy. Florfenicol and trimethoprim sulfadiazine were further selected to determine the minimum fungicidal concentration and the time needed to kill the fungus *in vitro*. Finally, for florfenicol, stability, toxicity and treatment efficacy in midwife toads (*Alytes muletensis*), experimentally infected with *B. dendrobatidis*, were determined.

## Methods

### Strains and culture conditions

Five *B. dendrobatidis* strains, kindly provided by Dr. J. Longcore, Dr. T. Garner and Dr. M. Fisher, were used in this study (Table 1). The strains were grown in TGhL broth (16 g tryptone + 2 g hydrolysed gelatin + 4 g lactose in 1 l distilled water) in 25 cm^2^ cell culture flasks at 20°C for 5 days. For the collection of zoospores for the experimental inoculation, one ml of a 5 day old broth culture was inoculated on TGhL agar (16 g tryptone + 2 g hydrolysed gelatin + 4 g lactose + 10 g agar in 1 l distilled water) and incubated for 5 to 7 days at 20°C. Zoospores were collected by flooding the agar with 2 ml of distilled water and collecting the supernatant.

**Table 1:** **Strains of *B. dendrobatidis *used in this study**

**Strain**	**Origin**
**JEL197**	*Dendrobatus azureus*
**JEL277**	*Ambystoma tigrinum*
**JEL310**	*Smilisca phaeota*
**JEL423**	*Lithobates catesbeianus*
**IA042**	*Alytes obstreticans*

### Determination of minimum inhibitory concentrations of antimicrobial agents

The minimum inhibitory concentration (MIC) of antimicrobial agents, each belonging to a different pharmacological group, for the *B. dendrobatidis* isolates were determined using a macrodilution method in 24 well plates following the method described by Martel et al. (2010) [8]. To each well, 200 μl of TGhL broth containing various concentrations of clindamycin (Sigma-Alderich Chemi Gmbh, Steinheim, Germany), doxycyclin (Sigma-Alderich Chemi Gmbh, Steinheim, Germany), enrofloxacin (Bayer B.V., Diegem, Belgium), florfenicol (20%), paromomycin (Sigma-Alderich Chemi Gmbh, Steinheim, Germany), polymyxin E (V.M.D., Arendonk, Belgium), sulfamethoxazole (Sigma-Alderich Chemi Gmbh, Steinheim, Germany), trimethoprim (Sigma-Alderich Chemi Gmbh, Steinheim, Germany), a combination of trimethoprim and sulfamethoxazole in a ratio of 1:5, a commercially available combination of trimethoprim and sulfadiazine in a ratio of 1:5 (trimazin 30%, Kela laboratoria nv, Hoogstraten, Belgium) or tylosin (Sigma-Alderich Chemi Gmbh, Steinheim, Germany), were added to 200 μl of a 3 to 4 day old growing culture containing approximately 10^5^*B. dendrobatidis* organisms consisting of a mixture of zoospores and zoosporangia. Addition of 1600 μl TGhL broth to each well, resulted in final assay antimicrobial concentrations of 8, 4, 2, 1, 0.5, 0.25, 0.125, 0.063, 0.031 and 0.016 μg/ml. In the two combinations of trimethoprim and sulfonamides this dilution resulted in final concentrations of trimethoprim/sulfonamide of 1.6/8, 0.8/4, 0.4/2, 0.2/1, 0.1/0.5, 0.05/0.25, 0.025/0.125, 0.013/0.063, 0.0063/0.031 and 0.0031/0.016 μg/ml. The *B. dendrobatidis* cultures were examined for visible growth at 1, 2, 4, 6, 8, 10 and 14 days of incubation. Growth was compared to wells, containing *B. dendrobatidis* in TGhL broth without antimicrobial compound and was defined both as microscopically visible development of zoospores to sporangia, and a visible increase in the number of fungal organisms. The MIC value was determined as the lowest drug concentration at which no growth of the *B. dendrobatidis* strain was visible after 14 days of incubation at 20°C using inverted microscopic examination. The experiment was carried out three times in triplicate for two strains (IA042 and JEL423). If growth inhibition was noticed for a given compound, the experiment was repeated for this compound for all five *B. dendrobatidis* strains (IA042, JEL197, JEL277, JEL310 and JEL423).

### Determination of the time to 100% killing of *B. dendrobatidis* by florfenicol and trimethoprim-sulfadiazin

Based on the results of the MIC determination, florfenicol and the commercially available combination of trimethorpim and sulfadiazine were selected for further testing. The time to 100% killing of *B. dendrobatidis* by florfenicol and trimethoprim - sulfadiazine (TMP-S) was assessed in 24 well plates as described previously [8]. For florfenicol, the strains IA042 and JEL423 were exposed to concentrations equal to the minimal inhibitory concentration (1 μg/ml), 10 times the minimal inhibitory concentration (10 μg/ml) and 100 times the minimal inhibitory concentration (100 μg/ml) during 1, 2, 4, 6, 8, 10 and 14 days. For TMP-S, the 2 strains were exposed to concentrations of 1.6, 3.2, 8 and 16 μg/ml trimethoprim in combination with 8 μg/ml, 16 μg/ml, 40 μg/ml and 80 μg/ml of sulfadiazine corresponding to 1, 2, 5 and 10 times the minimal inhibitory concentration of sulfadiazine respectively, during 1, 2, 4, 6, 8, 10 and 14 days. Growth in the wells was compared to that in untreated control wells, allowing estimation of the percentage of growth reduction. After the exposure time, the medium was replaced by fresh TGhL broth without antimicrobials and the plates were further incubated for 14 days at 20°C. The time to 100% killing at a given antimicrobial concentration was defined as the earliest time point of medium replacement at which no growth of the strain was observed after 14 days of incubation at 20°C. All experiments were carried out twice in triplicate.

### Killing capacity of florfenicol and trimethoprim - sulfadiazine towards *B. dendrobatidis* zoospores

To determine the killing capacity of florfenicol and trimethoprim sulfadiazine towards *B. dendrobatidis* zoospores of strain IA042, the uptake of propidium iodide (PI) after exposure to florfenicol or trimethoprim sulfadiazine was assessed using flow cytometry as described previously [8]. A suspension containing approximately 10^6^ zoospores/ml distilled water was exposed to either 1 μg/ml, 10 μg/ml or 100 μg/ml florfenicol or 1.6, 3.2, 8 or 16 μg/ml trimethoprim in combination with 8 μg/ml, 16 μg/ml, 40 μg/ml or 80 μg/ml sulfadiazine, and incubated for 1, 2 and 4 days at 20°C. The suspensions were transferred into Falcon tubes and PI was added to achieve a final concentration of 2 μg/ml PI. After incubation for 5 minutes at room temperature in the dark, the samples were analyzed using a FACSCanto flowcytometry system (Becton Dickinson Biosciences, Erembodegem, Belgium). Analyses were performed using FACSDiva software v5.0.1 (Becton Dickinson Biosciences). Vital zoospores were used to set light scatter gates for zoospore characteristics and zoospores killed with heat were used to set gates for PI positivity.

### Florfenicol stability in water

Based on the results obtained, florfenicol was selected for further experiments. To determine whether florfenicol treatment should include daily replacement of the bathing solution, the concentration of florfenicol was measured for 10 days in 20°C bathing solution containing either 10 μg/ml or 100 μg/ml florfenicol. Water concentrations of florfenicol were determined using a HPLC method with ultraviolet (UV) detection. The samples were analysed after appropriate dilution on a Thermo Separations Product (TSP, Fremont, CA, USA) HPLC-system using a Spectrasystem gradient pump, a Model AS 3000 autosampler and a Spectrafocus diode array detector set at 223 nm. A reversed-phase C_18_ column (100 x 4.6 mm ID, 5 μm Nucleosil, Varian, Harbor City, USA) and a guard column of the same type were used. The injection volume was 50 μl, the flow rate was 0.35 ml/min and the run time was set at 13 min. The mobile phase consisted of 80% water and 20% acetonitrile (VWR, Leuven, Belgium) [16]. All samples were taken in triplicate.

### Toxicity of florfenicol for tadpoles

Fifteen *Discoglossus scovazzi* tadpoles (Gosner stage 26–30) were individually housed in 600 ml of water at a temperature of 20°C in a room with a day-night cycle of 12 h:12 h and five animals were either exposed for 10 days to 10 μg/ml or 100 μg/ml florfenicol or not exposed to any antimicrobial drug. Animals were fed daily with commercial fish food pellets (Sera GmbH, Heinsberg, Germany) and weights were measured one hour before exposure and on day ten of this experiment. Daily observations for abnormal behavior, pathological signs or death were carried out. At the end of the experiment all animals were humanely euthanized and haematoxylin and eosin staining of paraffin embedded formalin fixed transversal whole body sections were examined for the presence of histological signs of toxicity. Statistical analyses of the results were performed using SPSS 17. This experiment was approved by the ethical committee of the Faculty of Veterinary Medicine, Ghent University.

### Treatment of experimentally infected midwife toads (*Alytes muletensis*) with florfenicol

#### *B. dendrobatidis* strain and growth condition

For the experimental infection, *B. dendrobatidis* strain IA042, isolated from a dead *Alytes obstetricans,* was used. The strain was grown on TGhL-agar for 5 days at 20°C. Subsequently, zoospores were collected by flooding the agar with 2 ml distilled water. Determination of zoospore density in the suspension was assessed using a Bürker counting chamber. The amount of zoospores in the suspension was then adjusted to approximately 10^7^ zoospores/ml.

#### Experimental animals

All animal experiments were approved by the ethical committee of the Faculty of Veterinary Medicine (Ghent University). Experiments were performed following all necessary ethical and biosecurity standards. Twelve captive bred *A. muletensis* at approximately 6 months post metamorphosis were used in this experiment. The animals were kept in filter top cages (32 x 17x 21 cm) lined with moist tissue, containing terracotta flower-pots as shelter and a petri-dish filled with dechlorinated tap water for bathing and were fed fruit flies with calcium supplementation *ad libitum*. Ambient temperature varied between 16-20°C and was monitored using an automatic data logger device (Escort intelligent mini 3.0 V data recorder, Escort Data Loggers Inc., Buchanan, VA, USA).

#### Experimental inoculation and treatment

The animals were inoculated three times with a four day interval by topical inoculation of 0.1 ml of the zoospore suspension per animal. To determine whether the experimental inoculation resulted in infection, samples of the drinking patch and plantar sides of the feet were collected using rayon tipped swabs (160 C, Copan Italia S.p.A., Brescia, Italy) and examined after 1 week for the presence of *B. dendrobatidis* DNA using the *q*PCR test described by Boyle et al. [17]. As soon as all animals tested positive for the presence of *B. dendrobatidis* DNA twice with an interval of 1 week, the animals were divided into two groups. The first group of six animals served as untreated positive controls. The second group of six animals was treated daily with florfenicol at a concentration of 10 mg/l water. The treatment consisted of transferring the frogs to a disinfected container lined with tissue paper and daily spraying of the frogs and the container contents with the respective solution. Rayon tipped swabs were used to collect samples from the pelvic region and toes of all animals from the first day after treatment onwards. These swabs were examined for the presence of *B. dendrobatidis* DNA using the *q*PCR method mentioned above.

## Results

### *B. dendrobatidis* is susceptible to florfenicol and sulfonamides

Growth inhibition was observed after exposure to florfenicol or sulfonamides but not clindamycin, doxycyclin, paromomycin, polymyxin-E, trimethoprim solely and tylosin. Florfenicol had the lowest MIC for *B. dendrobatidis* with three strains (IA042, JEL197 and JEL310) being susceptible to a concentration of 1 μg/ml and two strains (JEL277 and JEL423) to a concentration of 0.5 μg/ml. Inhibition of the development of the sporangia was visible at 4–5 days of exposure to these concentrations. For sulfamethoxazole, marked inhibition of sporangium development of *B. dendrobatidis-*strains IA042 and JEL423 was observed after 3–4 days of exposure to a concentration of 8 μg/ml but already at 2 days after exposure to 8 μg/ml when combined with 1.6 μg/ml trimethoprim. Visible growth of all 5 strains tested was inhibited by 1.6 μg/ml trimethoprim in combination with 8 μg/ml sulfamethoxazole or sulfadiazine after 2 days of exposure.

### Limited fungicidal activity of florfenicol and trimethoprim - sulfadiazine against *B. dendrobatidis*

Based on the results of the MIC determination, florfenicol and the combination trimethoprim sulfadiazine were selected to evaluate their fungicidal activity. At a concentration of 100 but not at 1 and 10 μg/ml of florfenicol, *B. dendrobatidis* cultures were completely killed within 14 days of exposure. At 10 μg/ml, sporadic viable zoospores were observed. For trimethoprim sulfadiazine, exposure to 16 μg/ml trimethoprim combined with 80 μg/ml sulfadiazine during 14 days was necessary to kill the *B. dendrobatidis* culture completely. Exposure to 3.2 and 8 μg/ml trimethoprim and 16 and 40 μg/ml sulfadiazine resulted in near absence of viable zoospores at 14 days. No significant killing was observed after exposure during 14 days to 1.6 μg/ml of trimethoprim combined with sulfadiazine at 8 μg/ml. No differences between the two tested strains (IA042 and JEL423) were observed. Compared to untreated zoospores, zoospores exposed to florfenicol at a concentration of 1, 10 or 100 μg/ml were not killed within 4 days, as assessed using flow cytometry. Exposure to trimethoprim sulfadiazine at a concentration of trimethoprim/sulfadiazine of 1.6/8 μg/ml, 3.2/16 μg/ml, 8/40 μg/ml or 16/80 μg/ml also did not kill zoospores within 4 days.

### Florfenicol remains stable during at least 10 days in tank water

The quantification of the florfenicol concentration in water samples using HPLC showed no decrease of the concentration during the ten day period at both concentrations (10 μg/ml and 100 μg/ml), rendering florfenicol suitable for a single bath treatment.

### Exposure of tadpoles to high doses of florfenicol results in decreased weight gain

#### Clinical observation

No clinical abnormalities were observed in tadpoles exposed to either 10 μg/ml or 100 μg/ml florfenicol.

#### Weights

Table 2 shows the average weights of the tadpoles at days 1 and 10 and the average weight gain during this ten day period. No significant differences were found between the weights of the different groups at the start of this experiment. Using one-way ANOVA and a Bonferroni test significant differences between means were found for weights at day 10 and mean weight gain between the group treated with 100 μg/ml and the control group. Also, weights at day 10 and mean weight gain significantly differed between the group treated with 100 μg/ml and the group treated with 10 μg/ml. No significant differences were found between the weights at day 10 and the mean weight gain between the group treated with 10 μg/ml florfenicol and the control group that did not receive florfenicol treatment. Histopathological examination of the tadpoles revealed the absence of lesions in the negative control animals and mild multifocal vacuolization of epithelial cells in gut and kidneys of the animals exposed to 10 μg/ml. In the tadpoles exposed to 100 μg/ml, diffuse and marked vacuolization of epithelial cells in the gut with marked apoptosis of enterocytes and mild to moderate vacuolization of the renal tubular cells were present.

**Table 2:** **Means of weights ± standard deviation at day 1 and day 10 and average weight gain ± standard deviation of *Discoglossus scovazzi *tadpoles (n = 5 per treatment group) during a ten day treatment experiment**

**Treatment**	**Weight (mg) day 1**	**Weight (mg)**	**Weight gain (mg)**
		**day 10**	
**Florfenicol 10 μg/ml**^**a**^	103 ± 12	197 ± 25	93 ± 24
**Florfenicol 100 μg/ml**^**b**^	115 ± 28	137 ± 10	22 ± 20
**Control**^**a**^	87 ± 20	210 ± 59	123 ± 57

### Fourteen day treatment with 10 μg/ml florfenicol reduces but does not eliminate *B. dendrobatidis* from experimentally infected postmetamorphic *Alytes muletensis*

**Figure 1:**
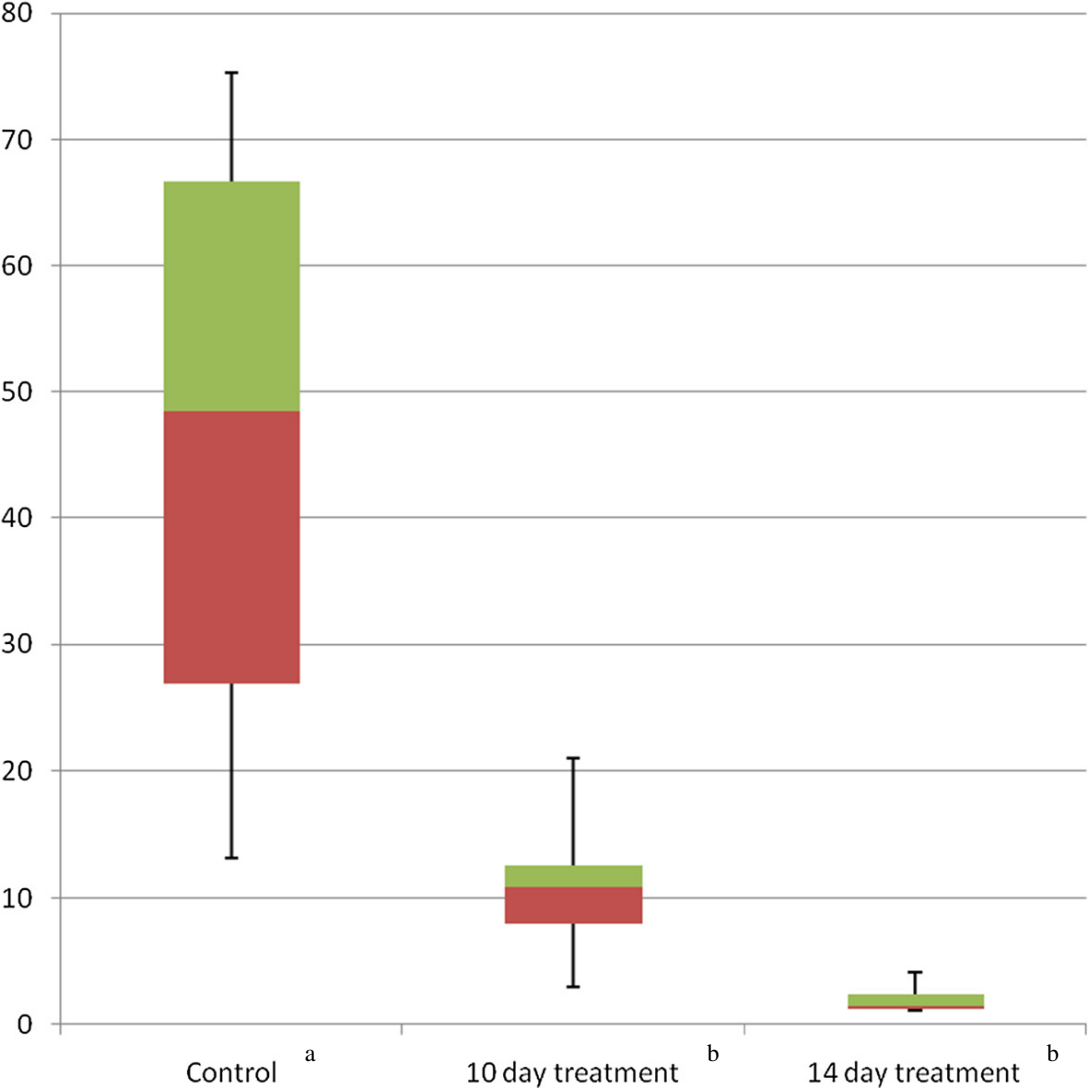
**Boxplots of genomic equivalents (GE) of *B. dendrobatidis *in skin swabs from *Alytes muletensis *that were either not treated (control, determined at the end of the 14 days treatment period) or treated during 10 days or 14 days with florfenicol at a concentration of 10 μg/ml.**

Immediately before the experimental treatment, all animals tested positive on *q*PCR with an average load of 5,4 ± 4,4 *B. dendrobatidis* genomic equivalents (GE) in skin swabs. After 14 days of treatment, all animals were still positive (Figure 1). The average load of genomic equivalents was lower in the treated than in the untreated animals. The Mann–Whitney U test showed statistically significant differences between both the 10 and 14 day treatment group and the control group (P < 0.011 and P < 0.006 respectively). No significant difference was found between the 10 and 14 day treatment groups. After treatment termination, increase in the number of GE in the treated frogs was noted (Table 3).

**Table 3:** **Mean load of genomic equivalents (GE) ± standard deviation of *B. dendrobatidis *in skin swabs collected from *Alytes muletensis *, experimentally inoculated with *B. dendrobatidis *, after a 14 days treatment period with 10 μg/ml florfenicol**

**Days post treatment**	**No. of animals**	**No. of positives**	**Genomic equivalents**
**1**	6	6	1.6 ± 1.5
**10**	6	6	5.1 ± 10.3
**24**	5	5	9.4 ± 7.1

## Discussion

In this study, a range of antimicrobial agents were tested for their *in vitro* efficacy against *B. dendrobatidis*. Only florfenicol, a chloramphenicol analog, sulfonamides and a combination of trimethoprim and sulfonamides showed *in vitro* efficacy against *B. dendrobatidis.* The use of antibiotics and chemotherapeutics against fungal diseases is not a new phenomenon. In previous studies, researchers found minocycline, polymyxin as well as doxycycline to inhibit growth in *Candida albicans* and *Candida tropicalis in vitro*[18-20]. *Fusarium* showed *in vitro* susceptibility to tobramycin and moxifloxacin [21]. Although trimethoprim *in se* had no visible effect on *in vitro* growth of *B. dendrobatidis*, the combination with sulfadiazine resulted in earlier growth arrest, which was clearly visible as impairment of the development of mature sporangia. Both agents thus have a synergistic effect, probably exerted on folic acid synthesis as shown in many prokaryotic taxa [22-24]. Although, the *in vitro* results obtained with trimethoprim - sulfadiazine might seem promising, not only this combination is not stable in water according to the manufacturer (Kela laboratoria nv, Hoogstraten, Belgium), we also observed acute toxicity in 2 *A. muletensis* exposed to a concentration of 16 μg/ml of TMP-S (data not shown). Indeed, acute toxicity of TMP-S has been reported for other animal species as well [25,26]. We therefore excluded TMP-S from further experiments. Because of the relatively low MIC value, the near absence of toxic effects at a concentration of 10 x the MIC value and its remarkable stability in water, florfenicol was selected for treatment of experimentally infected *A. muletensis*. Although a significant reduction of the number of *B. dendrobatidis* organisms was achieved, the treatment protocol did not eliminate the fungus from its amphibian host, resulting in a rebound effect post treatment. This finding emphasizes the importance of treatments that completely eliminate the fungus from the amphibian host. The reduction in zoospore count in skin samples in florfenicol treated animals found in this study, is similar to that found in the study using chloramphenicol (Bishop et al., 2009). Probably, this is due to the fungistatic activity of florfenicol, resulting only in growth inhibition. Indeed, both florfenicol and the combination trimethoprim sulfadiazine completely killed cultures of *B. dendrobatidis in vitro* at very high concentrations only. Skin defenses in amphibians appear not capable of clearing growth impaired *B. dendrobatidis* cells, which results in recrudescence of the infection after termination of treatment. Another explanation might be poor penetration of florfenicol in the anuran skin. Since clear toxicity symptoms were present in the highest treatment group, sufficient absorption is probably present. However, pharmacokinetic data of florfenicol in amphibians are lacking.

## Conclusions

In conclusion, the use of florfenicol applied in the bath water at 10 and 100 μg/l seems inappropriate to establish chytrid free populations in captivity. Also, the use of higher concentrations of florfenicol can be excluded because of the apparent toxicity at 100 μg/l. Further research with individual oral or parenteral treatments of florfenicol, longer treatment regimes and quantification of levels in skin tissue may be interesting, although impractical for treatment of a frog colony. However, since it is mandatory to eliminate *B. dendrobatidis* organisms completely from infected animals, application of voriconazole or itraconazole appears to be the only option at present to reliably control chytridiomycosis in infected amphibians.

## Competing interests

The authors declare that they have no competing interests.

## Authors contributions

MM carried out the *in vitro* as well as the *in vivo* studies, the statistical analyses and drafted the manuscript. AM participated in the design of the study and helped with the interpretation of results. PVR participated in both the *in vitro* and *in vivo* studies. KB and PDB carried out the HPLC tests. GV and RD participated in the pathological analyses of the toxicology studies. FV and FH participated in the design of the study and revised the manuscript. FP conceived of the study and participated in its design and coordination and helped to write the manuscript. All authors read and approved the final manuscript.
